# The complete mitochondrial genome of *Cladosporium anthropophilum* (cladosporiaceae, dothideomycetes)

**DOI:** 10.1080/23802359.2023.2167474

**Published:** 2023-01-25

**Authors:** Sung-Eun Cho, Ji Yeon Oh, Dong-Hyeon Lee

**Affiliations:** a Forest Biodiversity Division, Korea National Arboretum, Pocheon, South Korea; b Division of Forest Insect Pests and Diseases, National Institute of Forest Science, Seoul, South Korea

**Keywords:** Cladosporium anthropophilum, mitochondrial genome, pecan, plant pathogen

## Abstract

The genus *Cladosporium* (Cladosporiaceae, Capnodiales) is a large genus of Ascomycota. Although the genus is mostly reported as saprobes from a wide range of substrates with a worldwide distribution, members of this genus comprise infectious agents in animals and plants. Of those, *Cladosporium anthropophilum* is a common saprophytic fungus and has been found to be a human opportunistic pathogen and plant pathogen. The complete mitochondrial genome of *C. anthropophilum* is characterized through the *de novo* assembly of Illumina sequencing data. The mitochondrial genome is a circular molecule of 35,937 bp with 30.23% GC content and has a total of 47 genes including 16 protein-coding genes, 29 transfer RNA genes, and two ribosomal RNA genes. Based on protein-coding sequences of the mitochondrial genome sequence, a phylogenetic tree was constructed to demonstrate the phylogenetic relationship of *C. anthropophilum* and its related genera.

The ascomycete genus *Cladosporium* (Cladosporiaceae, Capnodiales) introduced by Link (Link 1816) is mainly known as a ubiquitous environmental saprobic fungus with a worldwide distribution and isolated from a wide range of substrates (Bensch et al. 2012). In some cases, the members of this genus have been reported as etiologic agents in vertebrate hosts, including humans and animals (Sandoval-Denis et al. 2015) and plant endophytes and pathogens causing leaf spots on diverse herbaceous and woody plants (Bensch et al. 2012). Of those, *Cladosporium anthropophilum* Sandoval-Denis et al. (2016) belongs to the *C. cladosporioides* species complex *and* is one of the most common saprophytic fungi given that it has been isolated quite frequently from a wide range of substrates including plant materials such as seeds or leaves (Tibpromma et al. 2019; Sandoval-Denis et al. 2016). The fungus can be further considered as a clinically relevant species given its prevalence from a set of clinical samples obtained from the USA (Sandoval-Denis et al. 2015).

An endophytic fungus, *Cladosporium anthropophilum,* was isolated from leaves of pecan from pecan orchards located in Miryang, South Korea (35°22′54.9″N 128°48′06.5″E). Of those successfully retained, the representative cultures were deposited to the culture collection (CDH) of the National Institute of Forest Science, South Korea (Accession No. CDH2021-02–05, nifos.forest.go.kr, Dong-Hyeon Lee, leedh2009@korea.kr) and a voucher specimen, CDH2021-03, was deposited to the Korean Agricultural Culture Collection (KACC), National Institute of Agricultural Sciences, South Korea (Accession No. KACC 49851, genebank.rda.go.kr). The DNA of the isolate CDH2021-03 was extracted from the fungal mycelium using CTAB DNA extraction method (Carter-House et al. 2020). Illumina paired-end (PE) library was constructed and sequenced using the Illumina HiSeqX platform with 151-bp PE reads. Raw sequencing data of 2.1 Gb were trimmed using the quality_trim program in CLC Assembly Cell package ver. 4.2.1 (QIAGEN, Denmark) with Phred scores > 20 and used for *de novo* assembly of mitochondrial genome according to the previous study (Lee et al. 2018, Cho et al. 2022). Trimmed high-quality read sequences of 1.8 Gb were *de novo* assembled using the clc_novo_assemble program with default parameters in the CLC Assembly Cell and then mitochondrial contigs were selected and ordered by similarity searches using BLAST against mitochondrial sequences from NCBI organelle genome resources (https://www.ncbi.nlm.nih.gov/genome/organelle/). The selected mitochondrial contigs were merged and gap-filled by a series of read mapping and extension to generate a complete and circularized mitochondrial genome. Sequence error was investigated and then corrected by read mapping of trimmed sequence data and manual curation. The complete mitogenome sequence was annotated using the GeSeq (Tillich et al. 2017) and Artemis (Carver et al. 2012) programs with reference mitochondrial genomes (GenBank accession nos. MN661341.1, MN657180.1, and MN657181.1). In addition, gene annotation was confirmed again by manual curation using BLAST searches against the reference mitochondrial genomes.

Mitochondrial genome of *C. anthropophilum* is a circular molecule of 35,937 bp with 30.23% GC content (GenBank accession number OK512878) and 1,640.6X mean coverage. A total of 47 genes were predicted in this mitochondrial genome, of which 16 are protein-coding genes including two free-standing open reading frames (ORFs), 29 transfer RNA genes, and two ribosomal RNA genes.

Phylogenetic analysis of *C. anthropophilum* with other taxa was performed using a Bayesian inference method with conserved 13 protein-coding sequences and revealed that *C. anthropophilum* is located in the same clade with other *Cladosporium* (Figure 1). Among *Cladosporium* species in the phylogenetic tree, *Cladosporium cladosporioides* was the closest to *C. anthropophilum*. Mitochondrial genome (MN661341) of *C. cladosporioides* is 36,768 bp in length and has 46 genes (Liu et al. 2020). Compared to *C. cladosporioides, C. anthropophilum* mitochondrial genome in this study is 831-bp smaller and has a similar number of genes except for one additional tRNA gene for tRNA-Thr. In this study, the phylogenetic inference of the genus *Cladosporium* and its related taxa was demonstrated, and this would help understand species evolution and phylogenetic relationships between the related taxa.

**Figure 1.**
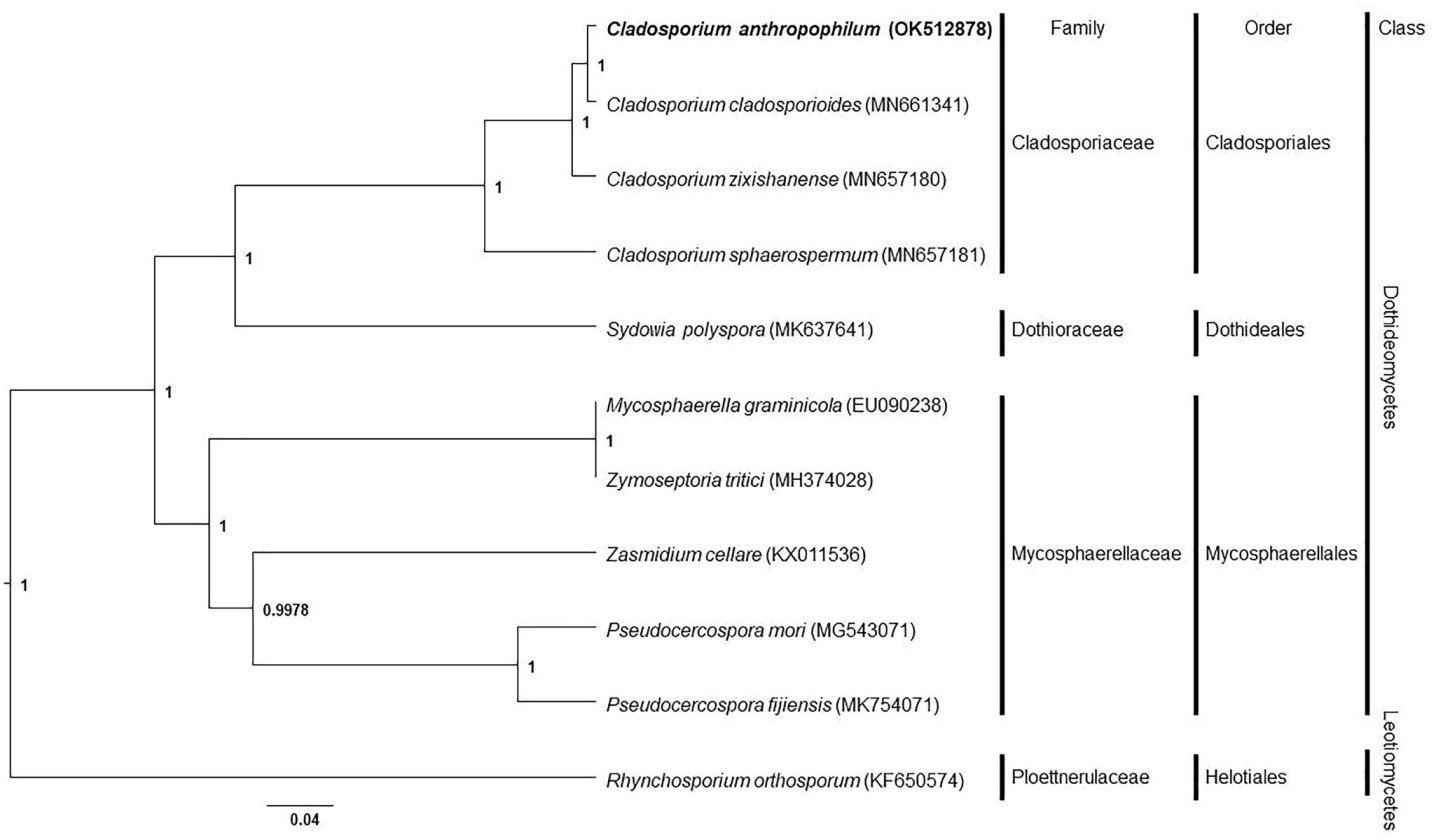
Phylogenetic tree of mitochondrial genomes of *Cladosporium anthropophilum* and its related species. Thirteen protein-coding sequences conserved in the mitochondrial genomes of 11 species were multiple-aligned using MAFFT (http://mafft.cbrc.jp/alignment/server/index.html) and used to generate phylogenetic tree using Bayesian inference method of BEAST2 (Bouckaert et al. 2019) with default parameter. Posterior probability values are on the branches. The isolate, *C. anthropophilum* (KACC 49851), obtained in this study was represented by bold letters. GenBank accession nos. of mitochondrial genome sequences used for this tree are indicated within parentheses.

## Data Availability

The genome sequence data that support the findings of this study are openly available in GenBank of NCBI at https://www.ncbi.nlm.nih.gov under the accession no. OK512878. The associated BioProject, SRA, and Bio-Sample numbers are PRJNA759657, SRR15693843, and SAMN21184963 respectively.

## Regulation statement

This study did not require any ethical approval and specific permissions or licenses to perform the research.
